# The *Yersinia pestis gcvB *gene encodes two small regulatory RNA molecules

**DOI:** 10.1186/1471-2180-6-52

**Published:** 2006-06-12

**Authors:** Sarah D McArthur, Sarah C Pulvermacher, George V Stauffer

**Affiliations:** 1 Department of Microbiology, The University of Iowa, Iowa City, Iowa 52242, USA

## Abstract

**Background:**

In recent years it has become clear that small non-coding RNAs function as regulatory elements in bacterial virulence and bacterial stress responses. We tested for the presence of the small non-coding GcvB RNAs in *Y. pestis *as possible regulators of gene expression in this organism.

**Results:**

In this study, we report that the *Yersinia pestis *KIM6 *gcvB *gene encodes two small RNAs. Transcription of *gcvB *is activated by the GcvA protein and repressed by the GcvR protein. The *gcvB*-encoded RNAs are required for repression of the *Y. pestis dppA *gene, encoding the periplasmic-binding protein component of the dipeptide transport system, showing that the GcvB RNAs have regulatory activity. A deletion of the *gcvB *gene from the *Y. pestis *KIM6 chromosome results in a decrease in the generation time of the organism as well as a change in colony morphology.

**Conclusion:**

The results of this study indicate that the *Y. pestis gcvB *gene encodes two small non-coding regulatory RNAs that repress *dppA *expression. A *gcvB *deletion is pleiotropic, suggesting that the sRNAs are likely involved in controlling genes in addition to *dppA*.

## Background

*Yersinia pestis *is the causative agent of plague, an infectious disease that results in lymphatic and blood infections [1]. The *Y. pestis *genome has been sequenced [2,3]. *Y. pestis *carries three plasmids of approximately 9.5, 70, and 100 kilobasepairs and each carries genes necessary for or that contribute to the pathogenicity of the bacterium [1]. The 70 kilobasepair plasmid encodes the low-calcium response stimulon (LCRS). Components of the LCRS include Yops (secreted anti-host proteins) and a type III secretion apparatus, or Ysc. The type III secretion apparatus is responsible for the translocation of the Yops to host cells that in turn down-regulate the response of the host phagocytic cells to infection [4]. Natural LCRS-negative mutants of *Y. pestis *occur, resulting in avirulence of the bacteria [1]. Besides the three plasmids, another pathogenicity factor is pigmentation. Cells of *Y. pestis *adsorb hemin at 26°C but not at 37°C and are pigmented (Pgm^+^) and virulent. Spontaneous nonpigmented (Pgm^-^) mutants of *Y. pestis *have been isolated. The Yersiniabactin iron transport system is part of the *pgm *locus, and its loss results in a Pgm^- ^mutant that is avirulent in mice unless hemin, ferrous sulfate, or ferric chloride is injected into mice along with the bacterial challenge [1].

Recently, a new class of molecules has been shown to regulate gene expression in bacteria, small non-coding regulatory RNAs (sRNAs). These sRNAs have gained much attention as recent genome-wide studies have identified sRNAs in a wide variety of organisms [5]. Most of these bacterial sRNAs are between 50 and 400 nucleotides (nts) in length and play important roles in global regulation [6,7]. Hfq is a small RNA binding protein and sRNAs in particular are targets for Hfq [6]. Binding of these sRNAs by Hfq in some way facilitates base pairing between the sRNAs and their respective target RNAs [8,9]. In *Vibrio cholerae*, sRNAs (Qrr RNAs) have been shown to regulate virulence genes [10] and in *Brucella abortus *an *hfq *mutation is lethal [9]. These results suggest that sRNAs and Hfq likely play important roles in the virulence of certain Gram-negative pathogens.

**Figure 1:**
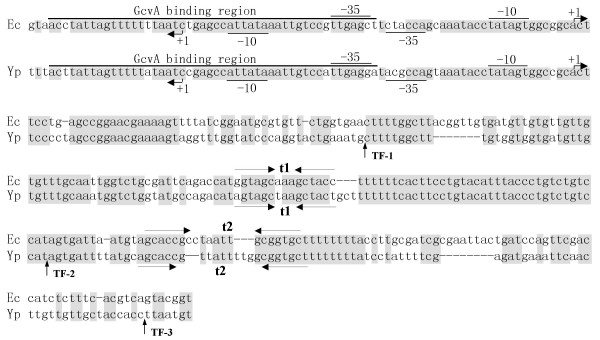
Comparison of the *E. coli *and *Y. pestis gcvA/gcvB *control regions and *gcvB *genes. Ec, *E. coli*; Yp, *Y. pestis*. Bases that are identical are boxed in gray. The *E. coli *promoter -10 and -35 sequences are underlined for *gcvA *[29] and overlined for *gcvB *[11]. Arrows indicate transcription start sites and directions of transcription of *gcvA *and *gcvB*. The GcvA binding region is indicated above the sequence [30]. The deduced *Y. pestis *-10 and -35 promoter sequences are underlined for *gcvA *and overlined for *gcvB*, and the deduced GcvA binding site is indicated above the sequence. Two Rho-independent transcription terminators for the *E. coli *and *Y. pestis gcvB *genes are indicated by inverted arrows. The fusion points for transcriptional fusions *gcvB*^+53^::*lacZ *(TF-1), *gcvB*^+164^::*lacZ *(TF-2) and *gcvB*^+251^::*lacZ *(TF-3) are indicated by vertical arrows.

The *E. coli gcvB *gene encodes sRNAs that are not translated *in vivo *[11]. A strain carrying a deletion of *gcvB *has constitutive synthesis of OppA and DppA, periplasmic binding proteins of the two major peptide transport systems normally repressed in cells grown in rich medium [12-14]. In addition to OppA and DppA, several other proteins were shown to increase or decrease in response to GcvB RNA levels, but the specific proteins were not identified [11]. Nevertheless, the results show that the GcvB RNAs are regulatory and possibly serve as global regulators. A computer search of the *Y. pestis *sequence showed that *Y. pestis *has a *gcvB *gene that shares considerable sequence homology with the *E. coli gcvB *sequence (Fig. 1). Thus, the GcvB RNAs from *E. coli *likely have functional counterparts in *Y. pestis*. The results of this study show that the *Y. pestis gcvB *gene encodes two sRNAs that, in turn, have regulatory activity. In addition, a deletion of the *gcvB *gene from the *Y. pestis *chromosome alters growth rate and colony morphology.

## Results and discussion

### Identification of the *Y. pestis gcvB *gene

**Figure 2:**
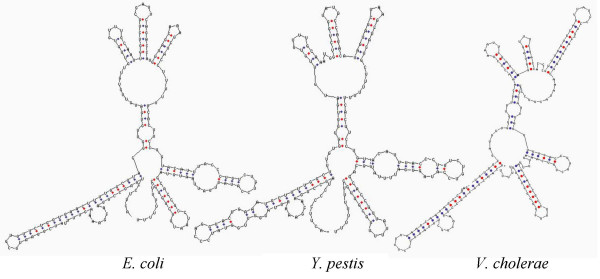
Secondary structures of GcvB RNAs with 77% (*Y. pestis*) and 53% (*V. cholerae*) identity to the *E. coli *GcvB RNA as predicted by the *mfold *algorithm [15].

The *E. coli gcvB *gene is divergently transcribed from *gcvA*, which encodes the activator protein for *gcvB *expression (Fig. 1) [11]. Thus, we used computer searches of genome sequences with the *gcvA *gene product as a query to predict *gcvB *homologs in other organisms. We identified GcvB-like RNA sequences in the genera *Yersinia*, *Salmonella*, *Haemophilus*, *Vibrio*, *Pasteurella*, *Shigella, Erwinia, Klebsiella, Photorhabdus *and *Actinobacillus*. Despite considerable sequence variation in many of these homologs, they are predicted by the *mfold *algorithm [15] to assume a similar secondary structure. A comparison of three of these GcvB RNAs is shown in Fig. 2. The location of the putative *Y. pestis gcvB *gene adjacent to and divergent from *gcvA*, its 77% sequence similarity to the *E. coli gcvB *sequence and its predicted secondary structure make it a likely homolog of *gcvB *in *Y. pestis*. Furthermore, identical *gcvB *sequences can be found in all other *Y. pestis *strains presently in the data base, both virulent and avirulent strains.

### The *Y. pestis gcvB *gene encodes two sRNAs

The *E. coli gcvB *gene encodes two sRNA transcripts that are not translated *in vivo *[11]. To determine if the *gcvB *gene in *Y. pestis *is functional and possibly encodes sRNAs, we initially constructed plasmid p*gcvB*^Yp+53^::*lacZ*, carrying a transcriptional fusion of the *gcvB *gene at basepair (bp) +53 to *lacZ*. Plasmid p*gcvB*^Yp+53^::*lacZ *and the vector alone were transformed into *Y. pestis *strain KIM6, the transformants grown in heart-infusion broth (HIB) + ampicillin (AP) to mid-log phase of growth and the cultures assayed for β-galactosidase activity. The KIM6 and KIM6 [pMC1403] control transformant gave 6 ± 0.3 and 6 ± 1 units of β-galactosidase activity, respectively, whereas the KIM6 [p*gcvB*^Yp+53^::*lacZ*] transformant gave 5,985 ± 118 units of β-galactosidase activity. The results suggest that the *gcvB *gene is expressed in *Y. pestis*.

The *gcvB *gene from *Y. pestis *possesses two possible Rho-independent transcription terminators, which if functional, would allow the production of two sRNAs of about 130 nts and 206 nts (Fig. 1). Three transcriptional gene fusions of the *Y. pestis gcvB *gene to *lacZ *were created to determine if these putative terminator sites function as transcription terminators *in vivo*. The three fusions, designated λ*gcvB*^Yp+53^::*lacZ*, λ*gcvB*^Yp+164^::*lacZ *and λ*gcvB*^Yp+251^::*lacZ*, were used to lysogenize *E. coli *strain GS162. The lysogens were then grown in Luria-Bertani broth (LB) [16] to mid-log phase of growth and the cultures assayed for β-galactosidase activity. About 44% of the β-galactosidase activity seen when the fusion precedes both terminators (λ*gcvB*^Yp+53^::*lacZ*) is lost when the fusion point follows terminator **t1 **(λ*gcvB*^Yp+164^::*lacZ*), implicating **t1 **as a site of transcription termination *in vivo *(Table 1). The remaining activity that escapes termination by **t1 **is not seen in GS162λ*gcvB*^Yp+251^::*lacZ*, indicating **t2 **also functions as a terminator *in vivo *(Table 1). When the 206 nts preceding terminator **t2 **for *gcvB *were analyzed, there were only short open reading frames (ORFs) that could encode polypeptides of 36 amino acids or less. These ORFs all lack good translational start sites and were not tested to determine if they encode small polypeptides. The *E. coli *GcvB RNAs are not translated into polypeptides [11]. Thus, we conclude that the products of the *gcvB *gene in *Y. pestis *are two sRNAs that are not translated, although the results do not completely rule out the possibility that the *Y. pestis *GcvB sRNAs encode small peptides. In *E. coli*, about 90% of the transcripts that initiate at the *gcvB *promoter terminate at terminator **t1**, and the remaining 10% terminate at terminator **t2 **[11]. A comparison of the *E. coli *and *Y. pestis ***t1 **sites shows that an additional 2 bps occur between the predicted GC-rich stem-loop structure and the run of T residues in the *Y. pestis ***t1 **site that are not present in the *E. coli *sequence, suggesting that the *Y. pestis ***t1 **site is likely less functional as a transcription terminator than the *E. coli ***t1 **site (Fig. 1). Nevertheless, the results are consistent with the *Y. pestis gcvB *gene encoding two sRNA molecules of about 130 and 206 nts and in roughly equal amounts.

**Figure 3:**
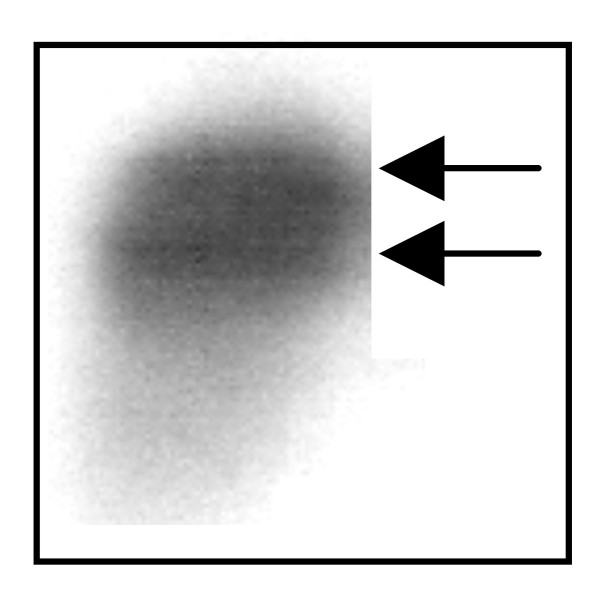
Northern blot analysis of GcvB from *Y. pestis *strain KIM6. Total cell RNA was isolated from strain KIM6 grown in HIB at 30°C to an O.D._600 _of 0.7 and probed with a ^32^P-labeled GcvB specific DNA probe as described in Methods. Two *gcvB *transcripts of about 206 and 130 nucleotides identified are indicated with arrows. Their sizes were determined based on their mobilities relative to the mobility of the *E. coli *GcvB RNA and 5S rRNAs (not shown).

We used Northern blotting to confirm that the *gcvB *locus in *Y. pestis *encodes sRNA transcripts of about 130 and 206 nts. Two small RNA molecules were detected in RNA isolated from *Y. pestis *KIM6 grown in HIB medium using a probe specific for the *gcvB *locus (Fig. 3). These results are consistent with the *in vivo *results with the *Y. pestis gcvB *transcriptional fusions.

### Regulation of the *Y. pestis gcvB *gene

The *E. coli gcvB *gene is activated by GcvA in the presence of glycine and repressed by GcvA + GcvR in its absence; this repression is enhanced by the addition of purines [11]. The regulation of the *Y. pestis gcvB *gene was tested with respect to the effects of glycine and purine supplementation to the growth medium and with respect to the GcvR and GcvA proteins and the GcvB RNAs. For these experiments we used the λ*gcvB*^Yp+53^::*lacZ *fusion to lysogenize appropriate *E. coli *host strains. The lysogens were grown in glucose minimal (GM) or GM supplemented with glycine or inosine to mid-log phase of growth and assayed for β-galactosidase levels. In the wild-type (WT) GS162λ*gcvB*^Yp+53^::*lacZ *lysogen, the addition of glycine to GM growth medium resulted in an 11.5-fold induction of β-galactosidase expression, whereas the addition of the purine inosine resulted in a 2.5-fold repression below the unsupplemented GM level (Table 2, line 1). In the *gcvA *mutant lysogen GS1118λ*gcvB*^Yp+53^::*lacZ*, the β-galactosidase levels were low and non-inducible by glycine (Table 2, line 2). The addition of inosine had no significant effect in the *gcvA *mutant strain. In the *gcvR *mutant lysogen GS1053λ*gcvB*^Yp+53^::*lacZ*, the β-galactosidase levels are constitutively high under all three growth conditions (Table 2, line 3). The results suggest that activation of the *Y. pestis gcvB *gene requires the GcvA protein and that repression requires the GcvR protein. Whether the negative regulation by GcvR requires a direct interaction of GcvR with GcvA as in *E. coli *[17,18] awaits further investigation. Furthermore, there appears to be no autoregulation of *gcvB *by its own sRNA products as the *gcvB *mutant lysogen GS1144λ*gcvB*^Yp+53^::*lacZ *shows normal regulation of the *gcvB*^Yp+53^::*lacZ *fusion (Table 2, line 4).

### *Y. pestis gcvA *encodes an activator protein for *gcvB *expression

Since activation of the *Y. pestis gcvB*^Yp+53 ^fusion in *E. coli *was dependent on GcvA (Table 2), we determined if the *Y. pestis gcvA *gene also encodes an activator protein for *gcvB *expression. We assumed this would be the case, as the *E. coli *and *Y. pestis *GcvA proteins are 88% identical at the amino acid sequence level. The *Y. pestis gcvA *gene was cloned into plasmid pACYC177 and tested for its ability to complement an *E. coli gcvA *mutant. The *E. coli *strain GS1132 carries a deletion of the *gcvA *gene [11]. This strain was lysogenized with an *E. coli *λ*gcvB*::*lacZ *transcriptional gene fusion and subsequently transformed with the control plasmid pACYC177, or pACYC177 carrying either the *E. coli *or the *Y. pestis gcvA *gene. The cells were grown in LB to mid-log phase of growth and assayed for β-galactosidase activity. As reported previously [11], expression of the *E. coli gcvB*::*lacZ *fusion was increased about 400-fold in the presence of the *E. coli gcvA *gene (Table 3, line 3). The *Y. pestis gcvA *gene also complemented the *E. coli *Δ*gcvA *strain, restoring *gcvB*::*lacZ *expression to nearly the same level as seen with the *E. coli gcvA *gene (Table 3, line 4). These results show that the *Y. pestis gcvA *gene codes for an activator protein capable of activating expression of an *E. coli gcvB*::*lacZ *fusion.

### The *Y. pestis gcvR *gene encodes a repressor protein for *gcvB *expression

Since deletion of the *gcvR *gene in *E. coli *results in constitutive expression of the *Y. pestis gcvB*^Yp+53 ^fusion (Table 2), we tested if the *Y. pestis gcvR *gene encodes a repressor for *gcvB *expression. We assumed this would be the case, as the *E. coli *and *Y. pestis *GcvR proteins are 75% identical at the amino acid sequence level. The *E. coli *strain GS1053 carries a Tn*10 *element inserted into the *gcvR *gene [19]. This strain was lysogenized with an *E. coli *λ*gcvB*::*lacZ*^+50 ^transcriptional gene fusion [11] and subsequently transformed with the control plasmid pACYC177, or pACYC177 carrying either the *E. coli gcvR *gene or the *Y. pestis gcvR *gene. The cells were grown in GM media to mid-log phase of growth and assayed for β-galactosidase activity. Expression of the *E. coli gcvB*::*lacZ *fusion is constitutive in the absence of a functional GcvR protein (Table 4, lines 1 and 2). The *gcvB*::*lacZ *fusion, however, was repressed in the presence of either pGS601, carrying *E. coli gcvR*, or p*gcvR*^Yp-p177^, carrying *Y. pestis gcvR *(Table 4, lines 3 and 4).

In *E. coli*, the GcvA and GcvR proteins interact to form a repressor complex [17,18]. The above results suggest that the *Y. pestis *GcvR protein interacts with the *E. coli *GcvA protein to form a repression complex. We tested if the *Y. pestis gcvA *and *gcvR *gene products also likely form a repressor complex to control expression of an *E. coli gcvB*::*lacZ *fusion. Strain GS1131λ*gcvB*::*lacZ *carries Δ *gcvR *Δ*gcvA *mutations. Strain GS1131λ*gcvB*::*lacZ *was transformed with plasmid p*gcvA*^Yp-p177^, p*gcvR*^Yp-p322^, or both plasmids. The vectors for p*gcvA*^Yp-p177 ^and p*gcvR*^Yp-p322 ^are pACYC177 and pBR322, respectively, to insure an excess of GcvR^Yp ^versus GcvA^Yp^. The cells were grown in GM media + appropriate antibiotics, harvested in mid-log phase of growth and assayed for β-galactosidase activity. The *Y. pestis gcvA *gene complemented the Δ*gcvA *mutation, resulting in activation of the *gcvB*::*lacZ *fusion (Table 4, line 7). The *Y. pestis gcvR *gene complemented the *gcvR *mutation, as repression of the *gcvB*::*lacZ *fusion occurred in the p*gcvA*^Yp-p177 ^p*gcvR*^Yp-p322 ^double transformant (Table 4, line 8). These results suggest that the GcvA and GcvR proteins likely interact to form a repression complex in *Y. pestis*. In *E. coli*, GcvA also activates the *gcvTHP *operon and GcvA + GcvR repress the operon [17,18]. Whether the *Y. pestis *GcvA and GcvR proteins also regulate the *Y. pestis gcvTHP *operon, or have additional regulatory roles, awaits further investigation.

### The *Y. pestis *GcvB RNAs regulate the *E. coli *and *Y. pestis dppA *genes

The *E. coli gcvB *gene negatively regulates the *dppA *and *oppA *genes [11]. In addition, many other genes were shown to be either negatively or positively regulated by the GcvB RNAs [11]. Thus, the *E. coli *GcvB RNAs are likely global regulators of gene expression. *Y. pestis *has homologs of *dppA *and *oppA*. To determine if the *Y. pestis *GcvB RNAs are regulatory, we transformed an *E. coli *Δ *gcvB *λ*dppA*::*lacZ *lysogen with p*gcvB*^Yp-p322^, the transformant and the parent lysogen were grown in LB to mid-log phase of growth and assayed for β-galactosidase levels. As expected, deletion of *gcvB *caused an increase in *dppA*::*lacZ *expression (Table 5, line 2). However, p*gcvB*^Yp-p322 ^complemented the *E. coli *Δ *gcvB *mutation, repressing the *E. coli dppA*::*lacZ *fusion (Table 5, line 3). Thus, the *Y. pestis *GcvB RNAs regulate the *E. coli dppA*::*lacZ *fusion. We then tested the regulatory activity of the GcvB RNAs in *Y. pestis *directly. A single-copy plasmid carrying a *Y. pestis dppA*::*lacZ *fusion was used to transform *Y. pestis *strain KIM6 and KIM6Δ *gcvB*. The transformants were grown in HIB + AP to mid-log phase of growth and assayed for β-galactosidase levels. Deletion of the *gcvB *gene resulted in a 7.3-fold increase in *dppA*::*lacZ *expression (Table 5, compare lines 4 and 5). The results suggest that the *Y. pestis *GcvB RNAs are regulatory molecules. However, the mechanism of GcvB RNA repression of *dppA *has not been determined. Although there is a region of 13–14 nucleotides in the *Y. pestis *GcvB RNA that can potentially base-pair with both the *E. coli *and *Y. pestis dppA *mRNAs near their ribosome binding sites, further studies are necessary to determine if base-pairing of GcvB RNA and *dppA *mRNA is part of the regulatory mechanism. Furthermore, in *E. coli*, the 206 nucleotide GcvB RNA is required for repression of *oppA *and *dppA *[11]. We are constructing a plasmid that will only produce the 130 nucleotide *Y. pestis *GcvB RNA to determine whether the 130 or 206 nucleotide RNA species is required for activity in *Y. pestis*.

### Deletion of the *Y. pestis gcvB *gene slows growth rate and alters colony morphology

**Figure 4:**
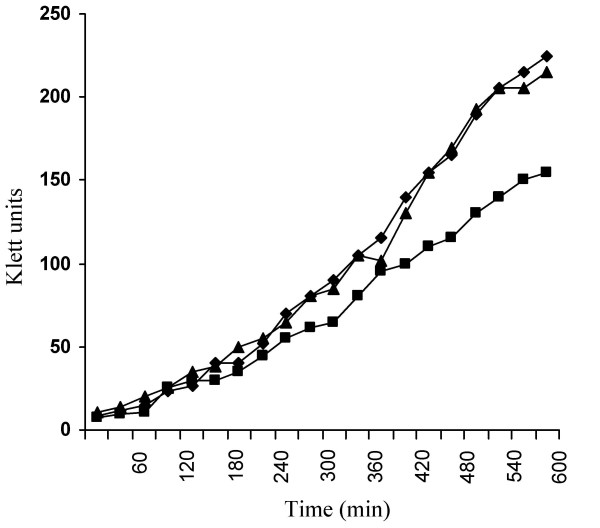
Effects of the Δ*gcvB *mutation on *Y. pestis *growth rates. *Y. pestis *strains KIM6 (◆), KIM6Δ*gcvB *(■), and KIM6Δ*gcvB*[p*gcvB*^Yp-sc^] (▲) were grown in HIB (+ AP for the p*gcvB*^Yp-sc ^transformant) at 37°C. The experiment was repeated three times. The curves show the results of a representative experiment.

The KIM6Δ *gcvB *strain routinely gave smaller colonies on HIB plates than the parent KIM6 strain. Thus, we investigated the growth of KIM6Δ *gcvB *to determine the effect of the Δ*gcvB *mutation on growth rate. The parent strain KIM6, KIM6Δ*gcvB *and KIM6Δ*gcvB *[p*gcvB*^Yp-sc^] were grown in HIB broth at 37°C. The generation times were then calculated. The KIM6 generation time at 37°C was 135 ± 15 minutes whereas KIM6Δ*gcvB *had a generation time of 194 ± 20 minutes (Fig. 4). The presence of p*gcvB*^Yp-sc ^in KIM6Δ*gcvB *complemented the *gcvB *deletion, as the generation time was reduced to 155 ± 8 minutes, close to the generation time of strain KIM6. Thus, deletion of the *gcvB *gene impairs the ability of *Y. pestis *to grow as well as the parent strain on either solid media or in liquid media. This is in contrast to *E. coli gcvB *deletion mutants that have no observable phenotype. The KIM6Δ *gcvB *strain also showed a different colony morphology from WT KIM6. WT KIM6 colonies appear smooth and sticky, whereas the KIM6Δ *gcvB *colonies appear dry and compact. The presence of p*gcvB*^Yp-sc ^in KIM6Δ*gcvB *again complemented the *gcvB *deletion, as the phenotype was restored back to the WT colony morphology.

In *E. coli*, many genes respond to the GcvB RNAs [11]. The pleiotropic nature of the *Y. pestis gcvB *deletion suggests that the *Y. pestis *GcvB RNAs are likely global regulators as well. Identification of the specific genes regulated by the GcvB RNAs that are responsible for the altered phenotype will allow us to test directly their involvement in virulence of the organism. In addition, the GcvB sequences and regulatory regions from bp -90 to +1, which include the putative GcvA binding sites for activation of *gcvB*, are 100% identical in all *Yersina pestis *strains, and greater than 92% identical in other *Yersinia *species. Thus, expression of *gcvB *and the regulatory mechanisms of the GcvB RNAs are likely similar in all *Yersinia *species.

## Conclusion

In summary, the *Y. pestis gcvB* gene is activated by the GcvA protein and repressed by the GcvR protein. The *gcvB *gene encodes two sRNAs that have regulatory activity, repressing *dppA *expression. A *gcvB *deletion is pleiotropic, suggesting that the GcvB RNAs possibly serve as global regulators in *Y. pestis*.

## Methods

### Bacterial strains, plasmids and phage

Bacterial strains, plasmids and phage used in this study are listed in Table 6 or are described in the text.

### Media

For *E. coli *strains, the complex medium used was LB [16] and the defined medium used was the minimal salts of Vogel and Bonner [20] supplemented with 0.4% glucose. GM media was always supplemented with 50 μg ml^-1 ^of phenylalanine and 1 μg ml^-1 ^of vitamin B1, since all *E. coli *strains carry *pheA, thi *mutations. Where indicated, glycine and inosine were added at 300 μg ml^-1 ^and 50 μg ml^-1^, respectively. For *Y. pestis *strains, HIB was used [21]. Agar was added at 1.5% to make solid media. Antibiotics were added at the following concentrations: AP, 150 μg ml^-1 ^for multi-copy plasmids and 50 μg ml^-1 ^for single-copy plasmids; chloramphenicol (CM), 20**μ**g ml^-1^; tetracycline (TC), 10 μg ml^-1^.

### β-galactosidase assays

β-galactosidase assays were performed on mid-log phase cells (OD_600_~0.5) as described by Miller [16]. Each experiment was repeated at least twice, with each sample assayed in triplicate.

### DNA manipulation

Plasmid DNA was isolated using Qiagen Miniprep kits as described by the manufacturer (Qiagen). Restriction enzyme digestions and DNA ligations were carried out according to the manufacturer (New England Biolabs). DNA sequencing was performed by the University of Iowa DNA Core Facility.

### PCR

PCR reactions were performed in 100 μl volumes. Each reaction mixture contained 10 μl 10 × polymerase buffer, 10 μl 10 × dNTPs (0.2 mM each), 5 μl *Y. pestis *DNA (~15 ng), 100 pmoles of forward and reverse primers designed specifically for each reaction, 1 μl of vent polymerase, and sterile water to bring the volume to 100 μl. PCR reactions were carried out under the following conditions: 5 min pre-incubation at 95°C, and then 30 cycles of 95°C for 30 sec, 45°C for 30 sec, and 72°C for 2 min.

### RNA extraction and Northern blot analysis

*Y. pestis *KIM6 was grown in HIB at 30°C to an O.D._600 _of 0.7, the cells collected for 1 minute in a microcentrifuge and immediately frozen at -70°C. Total cellular RNA was isolated using the MasterPure™ RNA purification kit (Epicenter). The final RNA pellet was re-suspended in water treated with diethyl pyrocarbonate and kept at -70°C. The RNA concentration was measured with a spectrophotometer at 260 nm. RNA (10 μg) was separated through a 1.5% formaldehyde gel and blotted on to a Biodyne Plus Membrane (ISC BioExpress). The blot was hybridized with a PCR generated DNA fragment from bp +1 to +198 of the *Y. pestis gcvB *gene and ^32^P-labeled using the RediprimeTM II Random Prime Labeling System (Amersham Biosciences). Hybridization of the blot was at 58°C as described [22].

### Construction of *gcvA, gcvB *and *gcvR *plasmids

The *Y. pestis gcvB *gene was cloned as follows. PCR primer YP-GCVB1F has an artificial *Eco*RI site and is complementary to the *Y. pestis *KIM6 DNA sequence beginning 114 bases upstream of the *gcvB *transcription start site. PCR primer YP-GCVB2R has an artificial *Hin*dIII site and is complementary to the *Y. pestis *DNA sequence beginning 45 bases downstream of the *gcvB *transcriptional termination site t2 (Fig. 1). Following PCR amplification, using *Y. pestis *chromosomal DNA as template, the amplified DNA was digested with *Eco*RI and *Hin*dIII, the 400 bp fragment carrying *gcvB *isolated from a 1% agarose gel and ligated into the *Eco*RI and *Hin*dIII sites of plasmid pBR322 [23], generating plasmid p*gcvB*^Yp-p322^. The *Y. pestis gcvA *and *gcvR *genes were cloned using a similar strategy. For *gcvA*, both the forward and reverse primers contained artificial *Hin*dIII sites complementary to the *Y. pestis *sequence beginning 111 bases upstream of the *gcvA *transcription start site and 349 bases downstream of the *gcvA *translation stop codon. For *gcvR*, both the forward and reverse primers contained artificial *Hin*dIII sites complementary to the *Y. pestis *sequence beginning 313 bases upstream of the *gcvR *transcription start site and 198 bases downstream of the *gcvR *translation stop codon. The PCR amplified fragments were cloned into the *Hin*dIII site of plasmid pACYC177 [24], generating plasmids p*gcvA*^Yp-p177 ^and p*gcvR*^Yp-p177^. In a second construct of *gcvR*, both the upstream and downstream primers contained artificial *Eco*RI sites and the PCR amplified fragment was cloned into the *Eco*RI site of plasmid pBR322, generating plasmid p*gcvR*^Yp-p322^. Each gene was sequenced at the University of Iowa DNA Core Facility to verify that no bp changes were introduced during the PCR amplification procedure.

### Construction of *lacZ *gene fusions

Three different transcriptional gene fusions of *gcvB *to the *lacZ *gene were constructed by PCR synthesis of fragments with common *Bam*HI termini 128 bp upstream of the *gcvB *transcription start site and 3 different fusion points within *gcvB*. In plasmid p*B*^Yp+53^::*lacZ*, the downstream PCR primer hybridized to the *gcvB *sequence beginning at bp +53 relative to the predicted transcription start site (+1) of *gcvB *(Fig. 1). A synthetic *Hin*dIII site was included at the end of the primer to allow the cloning of the 202 bp*Bam*HI-*Hin*dIII fragment into the *Bam*HI-*Hin*dIII sites of the *lacZ *transcriptional reporter plasmid pQF50 [25]. Plasmids p*B*^Yp+164^::*lacZ *and p*B*^Yp+251^::*lacZ *were constructed similarly except that the downstream primers used for PCR synthesis hybridized to the *gcvB *sequence beginning at bp +164 and +251 (Fig. 1), and the 313 and 400 bp fragments produced were cloned into the *Bam*HI-*Hin*dIII sites of pQF50. Each fusion was sequenced at the University of Iowa DNA Core Facility to verify that the fusions were at the correct sites and that no bp changes were introduced during the PCR amplification procedure. Each *gcvB *transcriptional fusion was then subcloned into plasmid pMC1403 [26], generating plasmids p*gcvB*^Yp+53^::*lacZ*, p*gcvB*^Yp+164^::*lacZ *and p*gcvB*^Yp+251^::*lacZ*, and subsequently transferred to phage λgt2 [27] as described [11], generating phage λ*gcvB*^Yp+53^::*lacZ*, λ*gcvB*^Yp+164^::*lacZ *and λ*gcvB*^Yp+251^::*lacZ*, respectively.

A single-copy *Y. pestis dppA*^Yp^::*lacZ *translational fusion was constructed in two steps. First, a *dppA*^Yp^::*lacZ *translational fusion was constructed using an upstream PCR primer with an *Eco*RI site complementary to the *Y. pestis *DNA sequence beginning 300 bps upstream of the *dppA *transcription initiation site and a downstream primer that contains an artificial *Sma*I site and that hybridizes to the *dppA *sequence after the 15^th ^codon relative to the translation initiation site. The 611 bp *dppA *fragment was cloned into the *Eco*RI and *Sma*I sites of the *lacZ *translational reporter plasmid pMC1403. The fusion was sequenced at the University of Iowa DNA Core Facility to verify that the fusion was at the correct site and that no bp changes were introduced during the PCR amplification procedure. The *dppA*^Yp^::*lacZ *fusion, along with the *lacY *and *lacA *genes, was then cloned into the single-copy plasmid pGS366, designated p*dppA*^Yp^::*lacZ*.

### Chromosomal deletion of *gcvB*

A *gcvB *deletion was constructed on the *Y. pestis *chromosome essentially as described [28]. *Y. pestis *strain KIM6 was transformed with plasmid pKD46, which encodes the Red recombinase of phage λ[28]. PCR products were then generated using two primers with 50 nt extensions that are complementary to sequences that flank the *gcvB *gene and 20 nt priming sequences that are complementary to the template plasmid pKD32 and that flank the CM^R ^gene and the FLP recognition sequence [28]. The PCR fragment was gel purified and used to transform *Y. pestis *KIM6 [pKD46]. The cells were plated on HIB plates with CM and CM^R ^recombinants were selected. One CM^R ^recombinant was single colony purified, chromosomal DNA was prepared, and PCR analysis was used to verify that the *gcvB *gene was deleted and replaced with the CM^R ^marker. The pKD46 plasmid is a temperature sensitive replicon and was cured by growth at 37°C [28]. The strain was designated KIM6Δ *gcvB*.

## Authors' contributions

SM carried out most of the genetic experiments and wrote the first draft of the manuscript. SP carried out the genetic experiments with *gcvR *and also performed the Northern analysis. GS carried out the computer search to identify putative *gcvB *genes in other organisms and was the principal investigator and supervised the project.

## Figures and Tables

**Table 1:** The *Y. pestis gcvB *gene encodes two sRNAs. Cells were grown in LB to an OD_600 _of ~0.5 and assayed for β-galactosidase activity [16]. Activity is expressed in Miller units.

Transformant	Relevant genotype	β-Galactosidase activity
GS162	WT	1 ± 0.1
GS162λ*gcvB*^Yp+53^::*lacZ*	WT	369 ± 40
GS162λ*gcvB*^Yp+164^::*lacZ*	WT	182 ± 7
GS162λ*gcvB*^Yp+251^::*lacZ*	WT	1 ± 0.3

**Table 2:** Regulation of the *Y. pestis gcvB*^+53^::*lacZ *transcriptional fusion in *E. coli*. Cells were grown in GM media with the indicated supplements to an OD_600 _of ~0.5 and assayed for β-galactosidase activity [16]. Activity is expressed in Miller units.

Lysogen	Relevant genotype	β-galactosidase activity for cells grown in:		
		GM	GM + glycine	GM + inosine
GS162λ*gcvB*^Yp+53^::*lacZ*	WT	15 ± 2	173 ± 2	6 ± 3
GS1118λ*gcvB*^Yp+53^::*lacZ*	Δ*gcvA*	2 ± 1	3 ± 1	2 ± 1
GS1053λ*gcvB*^Yp+53^::*lacZ*	*gcvR*	620 ± 58	419 ± 13	440 ± 174
GS1144λ*gcvB*^Yp+53^::*lacZ*	Δ*gcvB*	10 ± 2	140 ± 24	6 ± 2

**Table 3:** The *Y. pestis gcvA *gene encodes an activator protein. Cells were grown in LB to an OD_600 _of ~0.5 and assayed for β-galactosidase activity [16]. Activity is expressed in Miller units.

Lysogen	Relevant genotype	β-Galactosidase activity
GS1132λ*gcvB*::*lacZ*	Δ(*gcvA gcvB*)	<1
GS1132λ*gcvB*::*lacZ*[pACYC177]	Δ(*gcvA gcvB*)	<1
GS1132λ*gcvB*::*lacZ*[pGS335]	Δ(*gcvA gcvB*)/*gcvA*^Ec^	399 ± 22
GS1132λ*gcvB*::*lacZ*[p*gcvA*^Yp-p177^]	Δ(*gcvA gcvB*)/*gcvA*^Yp^	254 ± 21

**Table 4:** The *Y. pestis gcvR *gene complements an *E. coli gcvR *mutation. Cells were grown in GM media to an OD_600 _of ~0.5 and assayed for β-galactosidase activity [16]. Activity is expressed in Miller units.

Lysogen	Relevant genotype	β-Galactosidase activity
GS1053λ*gcvB*::*lacZ*	*GcvR*	308 ± 19
GS1053λ*gcvB*::*lacZ*[pACYC177]	*GcvR*	384 ± 175
GS1053λ*gcvB*::*lacZ*[pGS601]	*gcvR/gcvR*^Ec^	11 ± 1.5
GS1053λ*gcvB*::*lacZ*[p*gcvR*^Yp-p177^]	*gcvR*/*gcvR*^Yp^	14 ± 1.2
		
GS1131λ*gcvB*::*lacZ*	Δ*gcvA *Δ*gcvR*	2.2 ± 0.2
GS1131λ*gcvB*::*lacZ*[p*gcvR*^Yp-p322^]	Δ*gcvA *Δ*gcvR/gcvR*^Yp^	2.8 ± 0.1
GS1131λ*gcvB*::*lacZ*[p*gcvA*^Yp-p177^]	Δ*gcvA *Δ*gcvR/gcvA*^Yp^	393 ± 8
GS1131λ*gcvB*::*lacZ*[p*gcvA*^Yp-177 ^p*gcvR*^Yp-p322^]	Δ*gcvA *Δ*gcvR/gcvA*^*Yp *^*gcvR*^*Yp*^	6.8 ± 0.4

**Table 5:** Regulation of *E. coli *and *Y. pestis dppA*::*lacZ *translational gene fusions by the *Y. pestis gcvB *gene. Cells were grown in LB (*E. coli*) or in HIB (*Y. pestis*) at 37°C to an OD_600 _~0.5 and assayed for β-galactosidase activity [16]. Activity is expressed in Miller units. The parent strains KIM6 and KIM6Δ*gcvB *grown in HIB at 37°C showed <5 units of β-galactosidase activity.

Lysogen	Relevant genotype	β-Galactosidase activity
GS162λ*dppA*^Ec^::*lacZ*	WT	103 ± 24
GS1144λ*dppA*^Ec^::*lacZ*	Δ*gcvB*	554 ± 81
GS1144λ*dppA*^Ec^::*lacZ*[p*gcvB*^Yp-p322^]	Δ*gcvB/gcvB*^Yp^	154 ± 32
KIM6[p*dppA*^Yp^::*lacZ*]	WT	62 ± 13
KIM6Δ*gcvB*[p*dppA*^Yp^::*lacZ*]	Δ*gcvB*	455 ± 7

**Table 6:** Bacterial strains, plasmids and phage. All *E. coli *strains listed also carry Δ(*argF-lac*)*U169*, *pheA905*, *thi*, *araD129*, *rpsL150*, *relA1*, *deoC1*, *flb5301*, *ptsF25 *and *rpsR *mutations.

Strains/plasmids/phage	Relevant genotype	Source/reference
Strains*		
GS162	WT	This laboratory
GS1053	*gcvR*::Tn*10*	[19]
GS1118	Δ*gcvA*:Σ*aadA*	This laboratory
GS1131	Δ*gcvA*:Σ*aadA *Δ*gcvR*:ΣKN^R^	[11]
GS1132	Δ(*gcvA gcvB*):Σ*aadA*	[11]
GS1144	Δ*gcvB*:ΣCM^R^	This laboratory
KIM6	*lcr*^-^	[31]
KIM6Δ*gcvB*	Δ*gcvB*:ΣCM^R^	This study
Plasmid		
pGS366	Single-copy translational *lacZ *fusion vector	This laboratory
p*gcvB*^Yp-p322^	Carries *Y. pestis gcvB *in pBR322	This study
p*gcvB*^Yp-sc^	Carries *Y. pestis gcvB *in a single-copy vector	This study
p*gcvA*^Yp-p177^	Carries *Y. pestis gcvA *in pACYC177	This study
p*gcvR*^Yp-p177^	Carries *Y. pestis gcvR *in pACYC177	This study
p*gcvR*^Yp-p322^	Carries *Y. pestis gcvR *in pBR322	This study
p*dppA*^Yp^::*lacZ*	*Y. pestis dppA*::*lacZ *fusion in pGS366	This study
pGS335	Carries *E. coli gcvA *in pACYC177	This lab
pGS601	Carries *E. coli gcvR *in pACYC177	This lab
Phage		
λ*dppA*::*lacZ*	λgt2 with *E. coli dppA*::*lacZ *translational fusion	[11]
λ*gcvB*::*lacZ*	λgt2 with *E. coli gcvB*^+50^::*lacZ *transcriptional fusion	[11]
λ*gcvB*^Yp+53^::*lacZ*	λgt2 with *Y. pestis gcvB*^+53^::*lacZ *transcriptional fusion	This study
λ*gcvB*^Yp+164^::*lacZ*	λgt2 with *Y. pestis gcvB*^+164^::*lacZ *transcriptional fusion	This study
λ*gcvB*^Yp+251^::*lacZ*	λgt2 with *Y. pestis gcvB*^+251^::*lacZ *transcriptional fusion	This study

## References

[B1] Perry RD, Fetherston JD (1997). Yersinia pestis--etiologic agent of plague. Clin Microbiol Rev.

[B2] Deng W, Burland V, Plunkett G, Boutin A, Mayhew GF, Liss P, Perna NT, Rose DJ, Mau B, Zhou S, Schwartz DC, Fetherston JD, Lindler LE, Brubaker RR, Plano GV, Straley SC, McDonough KA, Nilles ML, Matson JS, Blattner FR, Perry RD (2002). Genome sequence of Yersinia pestis KIM. J Bacteriol.

[B3] Parkhill J, Wren BW, Thomson NR, Titball RW, Holden MT, Prentice MB, Sebaihia M, James KD, Churcher C, Mungall KL, Baker S, Basham D, Bentley SD, Brooks K, Cerdeno-Tarraga AM, Chillingworth T, Cronin A, Davies RM, Davis P, Dougan G, Feltwell T, Hamlin N, Holroyd S, Jagels K, Karlyshev AV, Leather S, Moule S, Oyston PC, Quail M, Rutherford K, Simmonds M, Skelton J, Stevens K, Whitehead S, Barrell BG (2001). Genome sequence of Yersinia pestis, the causative agent of plague. Nature.

[B4] Fields KA, Nilles ML, Cowan C, Straley SC (1999). Virulence role of V antigen of Yersinia pestis at the bacterial surface. Infect Immun.

[B5] Hershberg R, Altuvia S, Margalit H (2003). A survey of small RNA-encoding genes in Escherichia coli. Nucleic Acids Res.

[B6] Wassarman KM, Repoila F, Rosenow C, Storz G, Gottesman S (2001). Identification of novel small RNAs using comparative genomics and microarrays. Genes Dev.

[B7] Gottesman S (2005). Micros for microbes: non-coding regulatory RNAs in bacteria. Trends Genet.

[B8] Zhang A, Wassarman KM, Ortega J, Steven AC, Storz G (2002). The Sm-like Hfq protein increases OxyS RNA interaction with target mRNAs. Mol Cell.

[B9] Christiansen JK, Larsen MH, Ingmer H, Sogaard-Andersen L, Kallipolitis BH (2004). The RNA-binding protein Hfq of Listeria monocytogenes: role in stress tolerance and virulence. J Bacteriol.

[B10] Lenz DH, Mok KC, Lilley BN, Kulkarni RV, Wingreen NS, Bassler BL (2004). The small RNA chaperone Hfq and multiple small RNAs control quorum sensing in Vibrio harveyi and Vibrio cholerae. Cell.

[B11] Urbanowski ML, Stauffer LT, Stauffer GV (2000). The gcvB gene encodes a small untranslated RNA involved in expression of the dipeptide and oligopeptide transport systems in Escherichia coli. Mol Microbiol.

[B12] Olson ER, Dunyak DS, Jurss LM, Poorman RA (1991). Identification and characterization of dppA, an Escherichia coli gene encoding a periplasmic dipeptide transport protein. J Bacteriol.

[B13] Guyer CA, Morgan DG, Staros JV (1986). Binding specificity of the periplasmic oligopeptide-binding protein from Escherichia coli. J Bacteriol.

[B14] Manson MD, Blank V, Brade G, Higgins CF (1986). Peptide chemotaxis in E. coli involves the Tap signal transducer and the dipeptide permease. Nature.

[B15] Mfold algorithm [www.bioinfo.rpi.edu/applications/mfold/old/rna]. http://www.bioinfo.rpi.edu/applications/mfold/old/rna.

[B16] Miller JH (1992). A short course in bacterial genetics.

[B17] Ghrist AC, Heil G, Stauffer GV (2001). GcvR interacts with GcvA to inhibit activation of the Escherichia coli glycine cleavage operon. Microbiology.

[B18] Heil G, Stauffer LT, Stauffer GV (2002). Glycine binds the transcriptional accessory protein GcvR to disrupt a GcvA/GcvR interaction and allow GcvA-mediated activation of the Escherichia coli gcvTHP operon. Microbiology.

[B19] Ghrist AC, Stauffer GV (1995). Characterization of the Escherichia coli gcvR gene encoding a negative regulator of gcv expression. J Bacteriol.

[B20] Vogel HJ, Bonner DM (1956). Acetylornithinase of Escherichia coli: partial purification and some properties. J Biol Chem.

[B21] Kubota K, Yamamoto A (1966). [Tetanus toxin production. 1. Peptone-free medium for the toxin production with special reference to the significance of the bovine heart infusion]. Nippon Saikingaku Zasshi.

[B22] Song YJ, Stinski MF (2002). Effect of the human cytomegalovirus IE86 protein on expression of E2F-responsive genes: a DNA microarray analysis. Proc Natl Acad Sci U S A.

[B23] Bolivar F, Rodriguez RL, Greene PJ, Betlach MC, Heyneker HL, Boyer HW (1977). Construction and characterization of new cloning vehicles. II. A multipurpose cloning system. Gene.

[B24] Chang AC, Cohen SN (1978). Construction and characterization of amplifiable multicopy DNA cloning vehicles derived from the P15A cryptic miniplasmid. J Bacteriol.

[B25] Farinha MA, Kropinski AM (1990). Construction of broad-host-range plasmid vectors for easy visible selection and analysis of promoters. J Bacteriol.

[B26] Casadaban MJ, Chou J, Cohen SN (1980). In vitro gene fusions that join an enzymatically active beta-galactosidase segment to amino-terminal fragments of exogenous proteins: Escherichia coli plasmid vectors for the detection and cloning of translational initiation signals. J Bacteriol.

[B27] Panasenko SM, Cameron JR, Davis RW, Lehman IR (1977). Five hundredfold overproduction of DNA ligase after induction of a hybrid lambda lysogen constructed in vitro. Science.

[B28] Datsenko KA, Wanner BL (2000). One-step inactivation of chromosomal genes in Escherichia coli K-12 using PCR products. Proc Natl Acad Sci U S A.

[B29] Wilson RL, Stauffer GV (1994). DNA sequence and characterization of GcvA, a LysR family regulatory protein for the Escherichia coli glycine cleavage enzyme system. J Bacteriol.

[B30] Wilson RL, Urbanowski ML, Stauffer GV (1995). DNA binding sites of the LysR-type regulator GcvA in the gcv and gcvA control regions of Escherichia coli. J Bacteriol.

[B31] Sikkema DJ, Brubaker RR (1989). Outer membrane peptides of Yersinia pestis mediating siderophore-independent assimilation of iron. Biol Met.

